# Integrated mRNA and miRNA analysis reveals the regulatory network of oxidative stress and inflammation in *Coilia nasus* brains during air exposure and salinity mitigation

**DOI:** 10.1186/s12864-024-10327-w

**Published:** 2024-05-07

**Authors:** Jun Gao, Qi Mang, Yuqian Liu, Yi Sun, Gangchun Xu

**Affiliations:** 1 Key Laboratory of Freshwater Fisheries and Germplasm Resources Utilization, Freshwater Fisheries Research Center, Ministry of Agriculture, Chinese Academy of Fishery Sciences, Wuxi, Jiangsu 214081 China; 2 Wuxi Fisheries College, Nanjing Agricultural University, Wuxi, Jiangsu 214081 China; 3 College of Fisheries and Life Science, Shanghai Ocean University, Shanghai, 201306 China

**Keywords:** *Coilia nasus*, Air exposure, Salinity mitigation, miRNA-mRNA regulatory network, Inflammation

## Abstract

**Background:**

Air exposure is an inevitable source of stress that leads to significant mortality in *Coilia nasus*. Our previous research demonstrated that adding 10‰ NaCl to aquatic water could enhance survival rates, albeit the molecular mechanisms involved in air exposure and salinity mitigation remained unclear. Conversely, salinity mitigation resulted in decreased plasma glucose levels and improved antioxidative activity. To shed light on this phenomenon, we characterized the transcriptomic changes in the *C. nasus* brain upon air exposure and salinity mitigation by integrated miRNA-mRNA analysis.

**Results:**

The plasma glucose level was elevated during air exposure, whereas it decreased during salinity mitigation. Antioxidant activity was suppressed during air exposure, but was enhanced during salinity mitigation. A total of 629 differentially expressed miRNAs (DEMs) and 791 differentially expressed genes (DEGs) were detected during air exposure, while 429 DEMs and 1016 DEGs were identified during salinity mitigation. GO analysis revealed that the target genes of DEMs and DEGs were enriched in biological process and cellular component during air exposure and salinity mitigation. KEGG analysis revealed that the target genes of DEMs and DEGs were enriched in metabolism. Integrated analysis showed that 24 and 36 predicted miRNA-mRNA regulatory pairs participating in regulating glucose metabolism, Ca^2+^ transport, inflammation, and oxidative stress. Interestingly, most of these miRNAs were novel miRNAs.

**Conclusion:**

In this study, substantial miRNA-mRNA regulation pairs were predicted via integrated analysis of small RNA sequencing and RNA-Seq. Based on predicted miRNA-mRNA regulation and potential function of DEGs, miRNA-mRNA regulatory network involved in glucose metabolism and Ca^2+^ transport, inflammation, and oxidative stress in *C. nasus* brain during air exposure and salinity mitigation. They regulated the increased/decreased plasma glucose and inhibited/promoted antioxidant activity during air exposure and salinity mitigation. Our findings would propose novel insights to the mechanisms underlying fish responses to air exposure and salinity mitigation.

**Supplementary Information:**

The online version contains supplementary material available at 10.1186/s12864-024-10327-w.

## Introduction

Stress responses during breeding, fishing, and transportation can impact serum biochemical indexes, growth rate, and diminish antioxidant capacity and resistance in fish. Additionally, they can also disturb fluctuations in the concentration of glucose, lipid, and protein [1–3]. Air exposure is a highly stressful factor in fish. Previous studies have primarily concentrated on investigating the damage of air exposure, including oxidative stress levels [4], survival rates [5], energy metabolism [6], and hematological characteristics [7]. Presently, the emphasis in research on the effects of air exposure is primarily on exploring the mechanisms of damage caused, while the strategies to mitigate them have been paid less attention [6, 8]. However, it has been observed that properly adjusting the salinity of the aquatic environment is an effective strategy to alleviate stress in fish. Previous studies have demonstrated that altering salinity can enhance immune response and reduce serum glucose, plasma osmotic pressure, and antioxidant activity in rainbow trout (*Oncorhynchus mykiss*), threespot gourami (*Trichogaster trichopterus*), and *Labeo victorianus* during transportation stress [9–11]. Additionally, our own research has shown that adding 10‰ NaCl to aquatic water can positively alleviate stress responses to air exposure in *C. nasus* [12]. However, the precise mechanisms through which salinity alleviates stress are still not fully understood.

MicroRNAs (miRNAs) are a class of non-coding RNA molecules, typically consisting of 18–22 nucleotides. These mature miRNAs regulate target gene expression via binding to the 3′ untranslated region, leading to the silencing of those specific genes [13]. Previous studies have revealed that stress-related proteins, hormones, and cytokines can be impacted by various stressors in fish [14–16]. Recently, increasing attention was concentrated on dynamic alteration of miRNA expression during stress [17]. The increased expression of miR-101a was found in zebrafish (*Danio rerio*) under heat stress, and the induction of hsp70 was shown to impact the expression of miR-101a [18]. A total of 14 miRNAs exhibited significant expression changes in medaka (*Oryzias latipes*) under hypoxic stress. The mir-204-5p negatively regulated homeodomain-interacting protein kinase 1 (HIPK1), promoting apoptosis [19]. In Asian barramundi (*Lates calcarifer*), 25 immune-related miRNAs were significantly up-regulated in the spleen following a 24-hour exposure to lipopolysaccharide (LPS), and 52 miRNAs involved in 10 immune-related pathways [20]. The miR-122 was found to have pivotal roles in the metabolic pathway within the liver of true whitefish (*Coregonus lavaretus*) under microcystis stress. It achieved this by inhibiting target genes or modulating the phenotype of cellular genes, thereby regulating liver function [21]. With the in-depth study of miRNA functions, the significant roles played by miRNAs in stress response are gradually being unveiled. Exploring the functional role of miRNAs in stress response and mitigating stress in fish carries considerable scientific importance.

*Coilia nasus* is a rare species of *Coilia* known for its strong stress response, usually leading to high mortality. Previous studies on *C. nasus* have demonstrated that oxidative stress, apoptosis, and elevated mortality rates were induced by transport stress [22]. The addition of salt before and after air exposure has been found to have a positive effect on oxidative stress, endoplasmic reticulum stress, and apoptosis in *C. nasus* juveniles [12]. However, previous investigations have primarily focused on the transcriptional level and have not addressed the underlying regulatory mechanisms. Therefore, in our present study, we conducted microRNA sequencing and integrated RNA-seq analysis to elucidate the regulatory mechanisms in *C. nasus*. Our findings will propose novel insights to stress mitigation in fish and promote healthier aquaculture practices.

## Results

### Alteration of biochemical indexes during air exposure and salinity mitigation

**Fig. 1:**
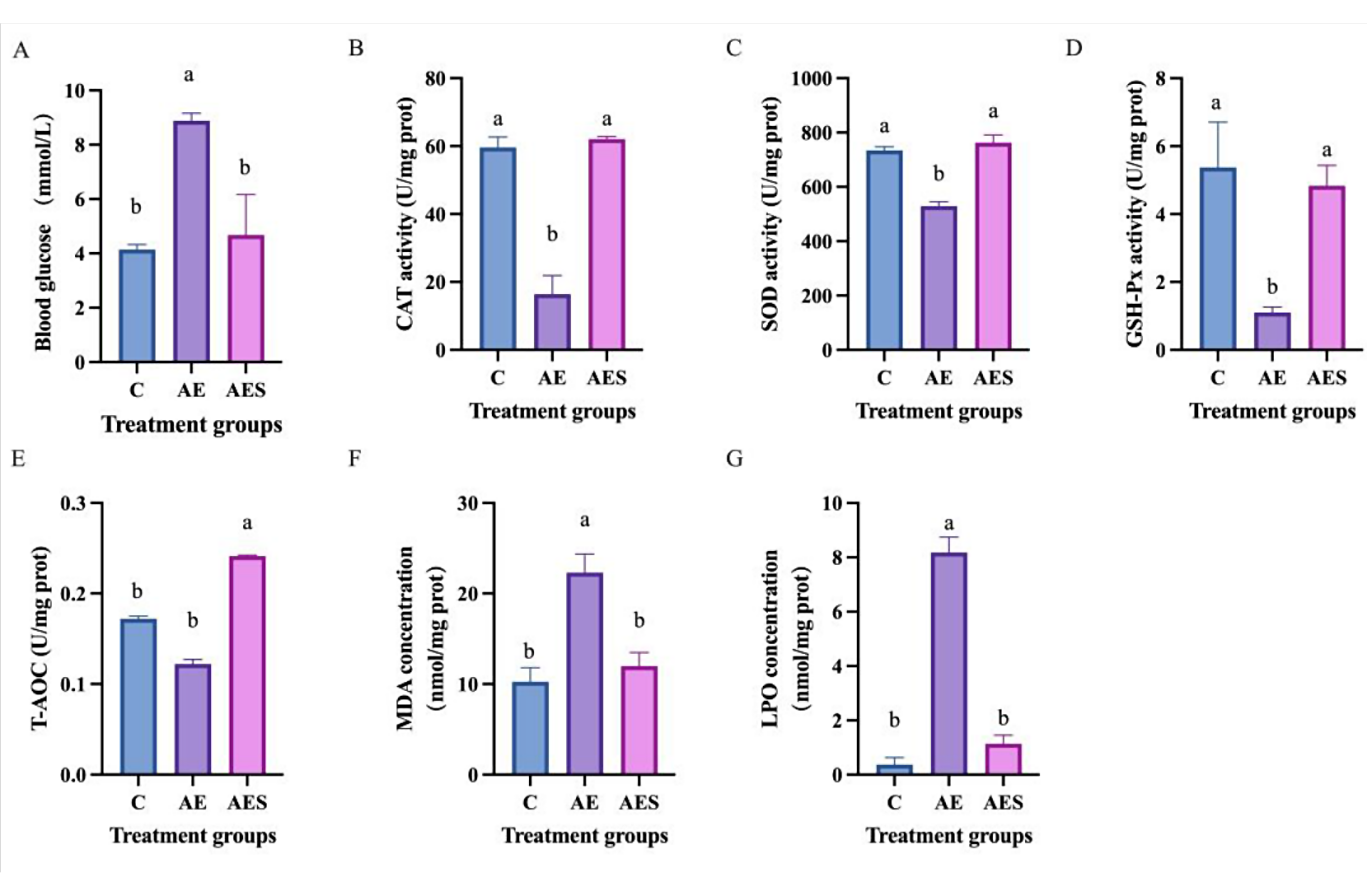
Effects of air exposure and salinity mitigation on glucose (**A**) in serum, CAT (**B**), SOD (**C**), GSH-Px (**D**), T-AOC (**E**), MDA (**F**), and LPO (**G**) in brain. The results were showed in means ± SD. Different letters indicate significant difference at *P* < 0.05

The results of biochemical indexes in serum during air exposure and salinity mitigation were showed in Fig. 1. Compared with C group, the concentrations of glucose, malondialdehyde (MDA), and lipid peroxidation (LPO) were significantly increased in AE group (*P* < 0.05), while the catalase (CAT), superoxide dismutase (SOD), and glutathione peroxidase (GSH-Px) activities were significantly decreased in AE group (*P* < 0.05). Moreover, compared with AE group, the concentrations of glucose, MDA, and LPO were significantly decreased in AES group (*P* < 0.05), while CAT, SOD, GSH-Px activities, and total antioxidant capacity (T-AOC) were significantly increased in AES group (*P* < 0.05).

### miRNA expression profiles during air exposure and salinity mitigation

A total of 12,051,985 to 22,516,624 clean reads were obtained by removing adapters, filtering out low-quality sequences, and eliminating contamination (Table S1). The length distribution of the small RNAs in each library ranged from 19 to 22 nucleotides, with the peak distribution observed at 22 nucleotides (Figure S1). The obtained small RNAs were annotated using the Rfam database to remove known non-miRNA sequences such as rRNA, snoRNA, snRNA, tRNA, and others (Table S2). Between 87.64% and 96.39% of the filtered small RNA sequences from each group were successfully mapped to the *C. nasus* genome (GCA_007927625.1) (Table S3). In total, we identified 1,435 known miRNAs and 604 novel miRNAs using Bowtie, RNAfold, and mireap based on the miRBase database. A total of 629 DEMs were identified between the AE group and C group, with 64 DEMs being up-regulated and 565 DEMs being down-regulated. Between the AES group and AE group, 429 DEMs were identified, including 294 up-regulated DEMs and 135 down-regulated DEMs. Additionally, between the AES group and C group, 692 DEMs were obtained, consisting of 205 up-regulated DEMs and 487 down-regulated DEMs.

### GO and KEGG classification analysis of DEMs’ target genes

**Fig. 2:**
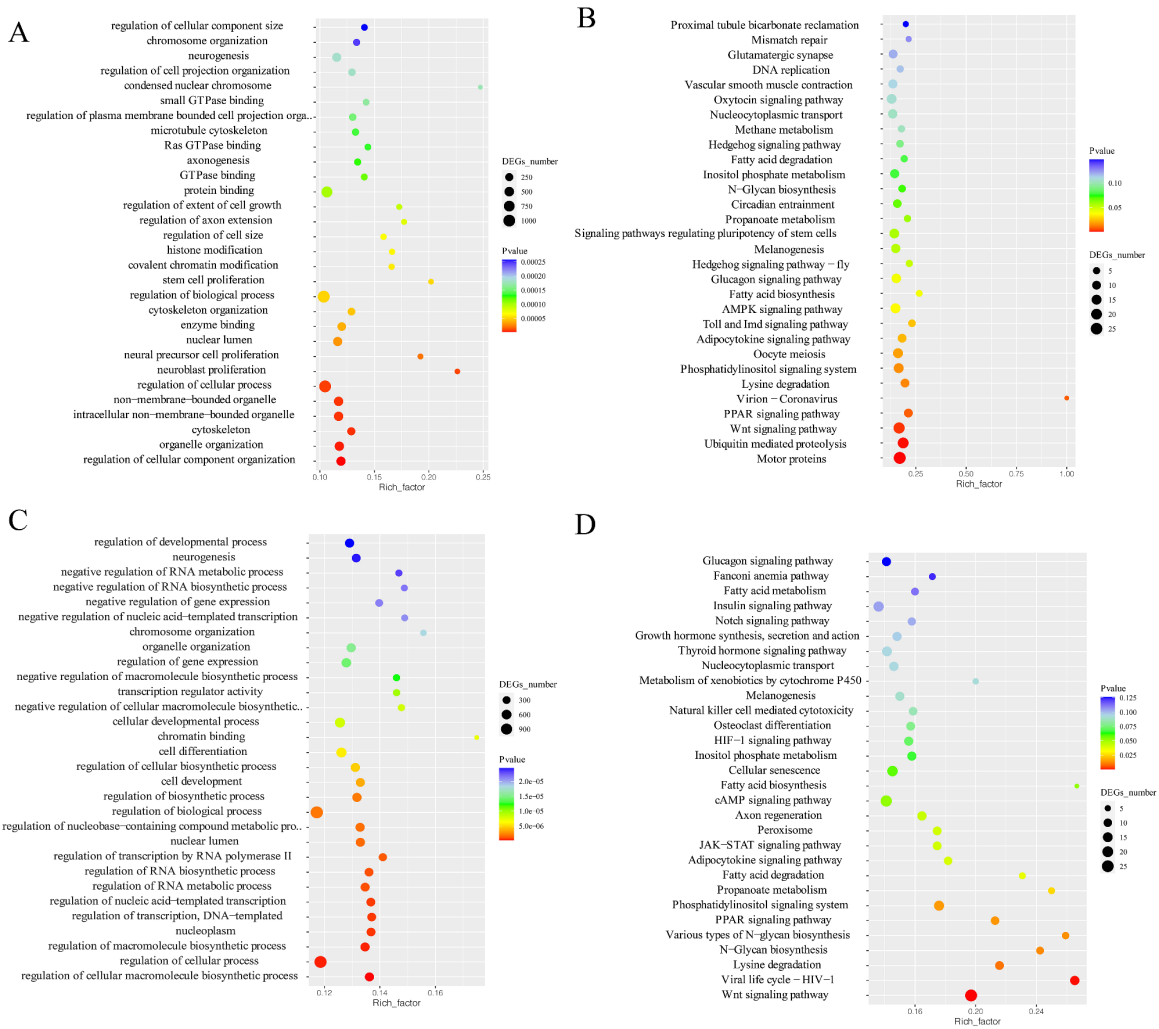
Functional enrichment of differentially expressed miRNAs (DEMs) during air exposure and salinity mitigation. GO enrichment (**A**) and KEGG enrichment (**B**) of DEMs between AE and C group. GO enrichment (**C**) and KEGG enrichment (**D**) of DEMs between AES and AE group

Comparing the AE group with the C group, the GO analysis demonstrated that the target genes of DEMs were categorized into biological process (regulation of cellular component organization, organelle organization) and cellular component (cytoskeleton, non-membrane-bounded organelle, intracellular non-membrane-bounded organelle) (Fig. 2A); KEGG analysis demonstrated that the target genes of DEMs were significantly enriched in pathways such as the Motor proteins, Ubiquitin mediated proteolysis, Wnt signaling pathway, and PPAR signaling pathway (Fig. 2B). In the comparison between the AES group and AE group, the GO analysis revealed that the target genes of DEMs were enriched in biological process (regulation of cellular macromolecule biosynthetic process, regulation of cellular process, regulation of macromolecule biosynthetic process, regulation of transcription, DNA-templated) (Fig. 2C); KEGG analysis showed significant enrichment of target genes in Wnt signaling pathway, PPAR signaling pathway, Lysine degradation, and N-Glycan biosynthesis (Fig. 2D).

### Transcriptomic expression profiles during air exposure and salinity mitigation

To investigate the genes related to air exposure and salinity mitigation, a total of nine samples from the brains of *C. nasus* were used to construct three groups of cDNA libraries. These groups consisted of control groups (C), air exposure groups (AE), and salinity mitigation groups (AES). Initially, 46,571,410 to 51,277,748 raw reads were generated. 46,190,474 to 50,859,056 clean reads remained via removing adapters, filtering low-quality sequences, and eliminating contamination (Table S4). Furthermore, 67.16–71.22% of the clean reads from each group were successfully mapped to the *C. nasus* genome (Table S5). A total of 791 differentially expressed genes (DEGs) were identified between the AE group and C group, consisting of 383 up regulated DEGs and 408 down regulated DEGs. Similarly, between the AES group and AE group, 1016 DEGs were identified, comprising 473 up regulated DEGs and 543 down regulated DEGs. Additionally, between the AES group and C group, 1651 DEGs were identified, with 572 up regulated DEGs and 1079 down regulated DEGs.

### GO and KEGG classification analysis of DEGs

**Fig. 3:**
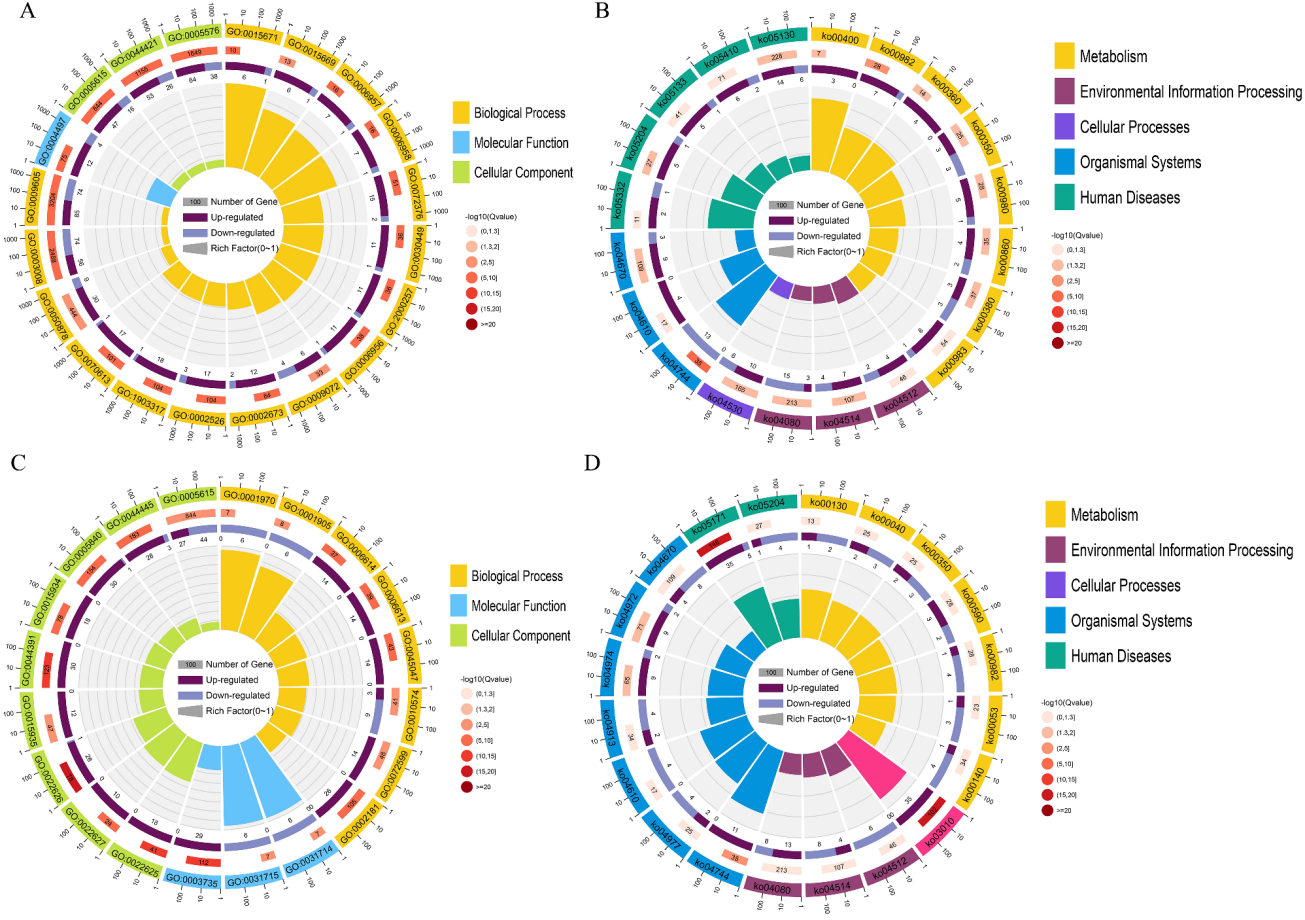
Functional enrichment of differentially expressed genes (DEGs) during air exposure and salinity mitigation. GO enrichment (**A**) and KEGG enrichment (**B**) of DEGs between AE and C group. GO enrichment (**C**) and KEGG enrichment (**D**) of DEGs between AES and AE group. Details of GO and KEGG terms were listed on Table S6

Between the AE group and C group, the GO analysis revealed DEGs were categorized into biological process (protein activation cascade and regulation of protein maturation) (Fig. 3A); KEGG analysis demonstrated that DEGs were enriched in metabolism (Amino acid metabolism and Xenobiotics biodegradation and metabolism) (Fig. 3B). Between the AES group and AE group, GO analysis showed that DEGs were enriched in biological process (cotranslational protein targeting to membrane and protein targeting to ER) and cellular component (cytosolic ribosome and ribosomal subunit) (Fig. 3C); KEGG analysis showed that the target genes of DEMs were enriched in metabolism (Carbohydrate metabolism and Amino acid metabolism) and organismal systems (Sensory system and Digestive system) (Fig. 3D).

### Integrated analysis of the miRNA-mRNA-pathway regulatory network

**Fig. 4:**
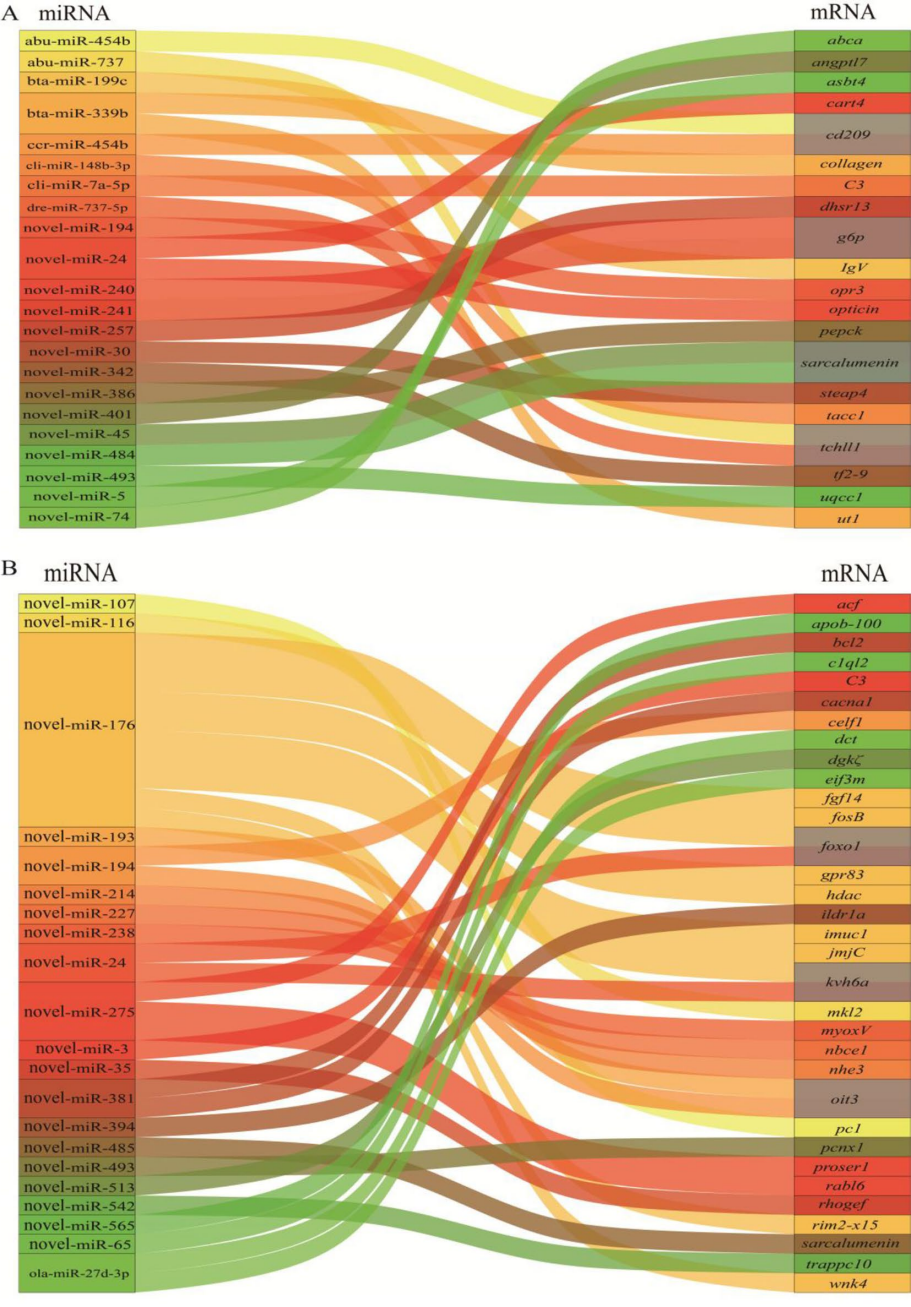
miRNA-mRNA regulatory networks potentially involved in air exposure and salinity mitigation

**Fig. 5:**
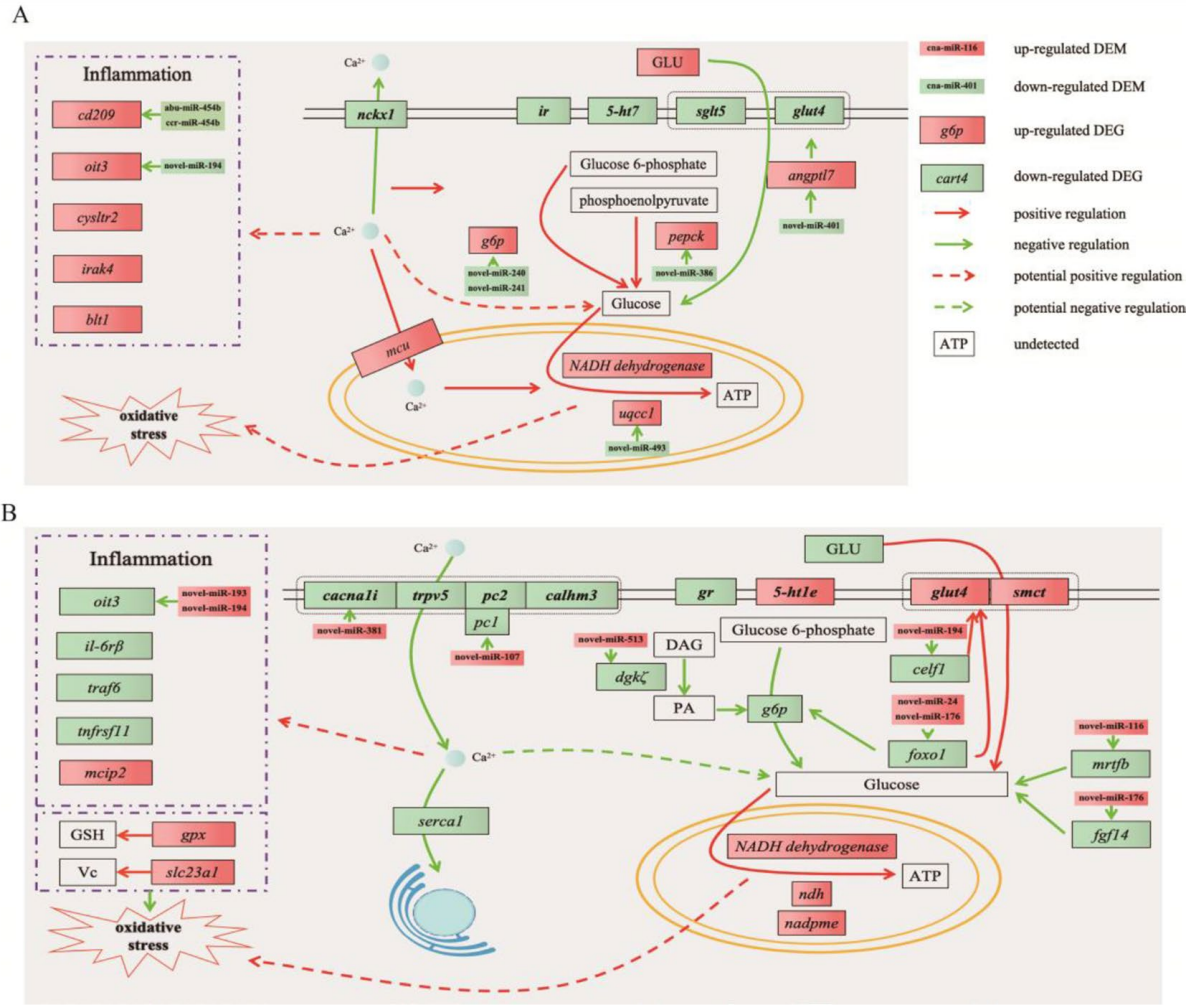
Schematic diagram of predicted molecular mechanism during air exposure and salinity mitigation in brains of *C. nasus*. (**A**) key DEMs and DEGs engaged in brains of *C. nasus* during air exposure. (**B**) key DEMs and DEGs engaged in brains of *C. nasus* during salinity mitigation. The full names and expression profiles of genes were listed in Table S8

Based on the obtained expression profiles of mRNA and miRNA, differentially expressed miRNAs (DEMs) were identified along with their target differentially expressed genes (DEGs) showing a significantly negative correlation. The potential regulatory relationships between them were illustrated in Fig. 5. Through integrated analysis, it was determined that 20 DEGs between the AE and C group were negatively regulated by 22 DEMs, resulting in 24 miRNA-mRNA pairs (Fig. 4A) (Table S6). Similarly, 33 DEGs between the AES and AE group were found to be negatively regulated by 21 DEMs, generating 36 miRNA-mRNA pairs (Fig. 4B) (Table S7).

Taking into account comprehensive bioinformatic analysis, potential predictions of regulatory relationships, and literature searches, a putative schematic diagram was compiled to depict the essential miRNAs and mRNAs involved in glucose metabolism, Ca^2+^ transport, inflammation, and oxidative stress in the brains of *C. nasus* during air exposure (Fig. 5A) and salinity mitigation (Fig. 5B).

### Validation of miRNA and mRNA expression level

**Fig. 6:**
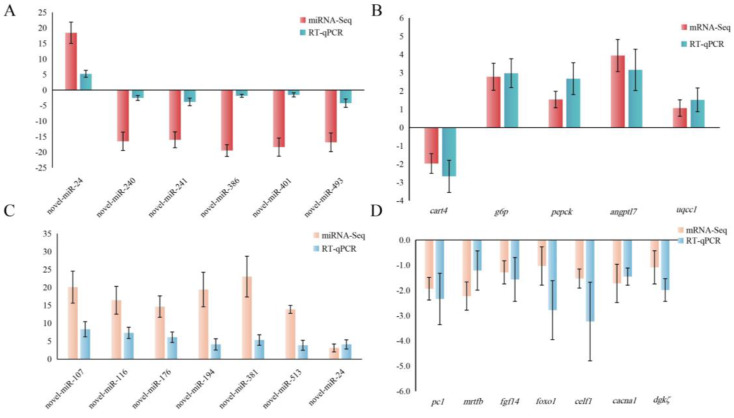
RT-qPCR validation in the brains of *C. nasus*. (**A**) RT-qPCR validation of 6 DEMs in AE vs. C. (**B**) RT-qPCR validation of 5 DEGs in AE vs. C. (**C**) RT-qPCR validation of 7 DEMs in AES vs. AE. (**D**) RT-qPCR validation of 7 DEGs in AES vs. AE. The results were showed in means ± SD.

**Fig. 7:**
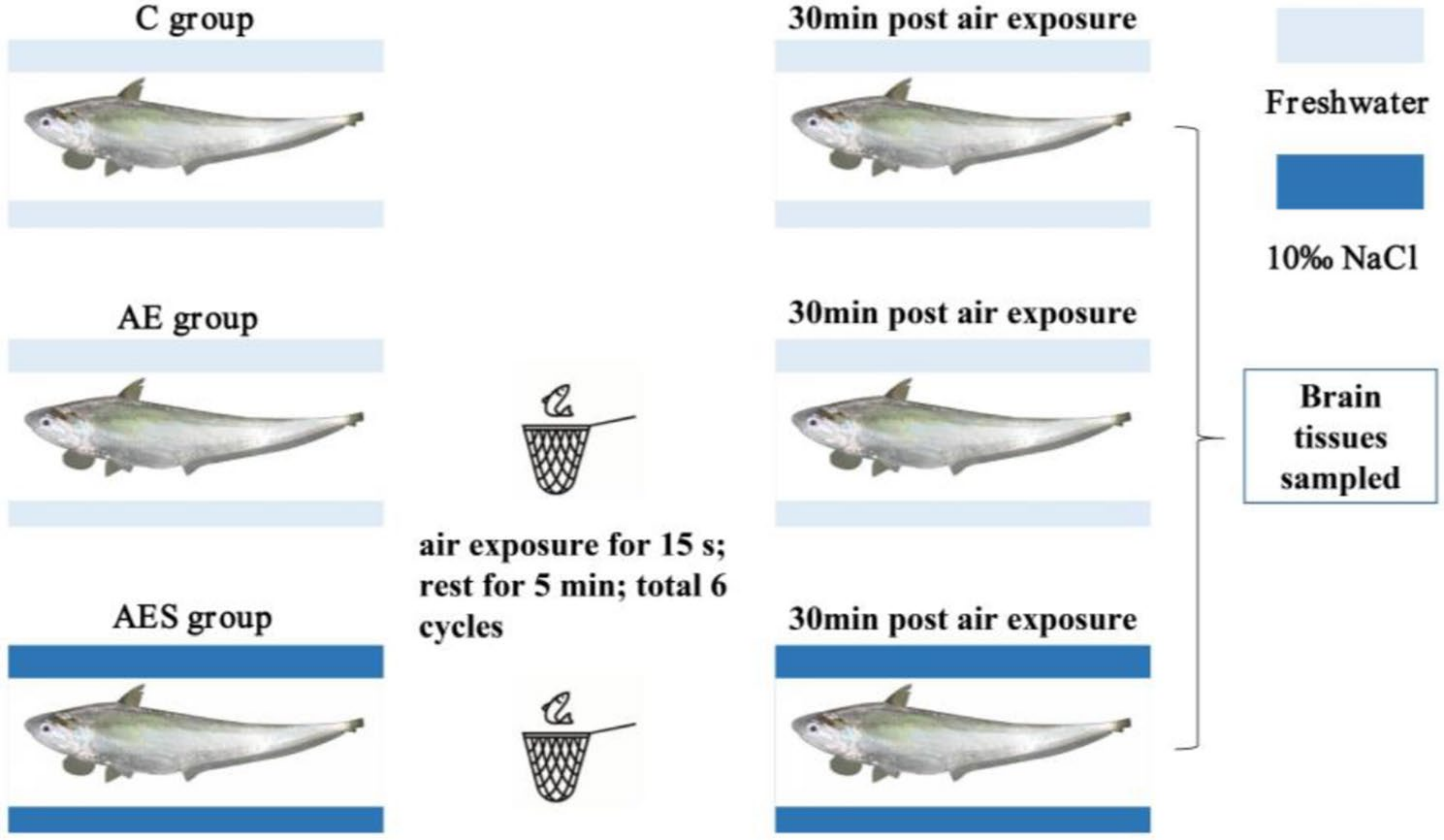
The experimental protocol of the present study

To validate the accuracy of the small RNA-Seq and RNA-Seq data, the expression levels of 6 differentially expressed miRNAs (DEMs) (novel-miR-24, novel-miR-240, novel-miR-241, novel-miR-386, novel-miR-401, and novel-miR-493) (Fig. 6A) and 5 differentially expressed genes (DEGs) (*cart4*, *g6p*, *pepck*, *angptl7*, and *uqcc1*) (Fig. 6B) from the identified miRNA-mRNA pairs between the AE and C group were measured using RT-qPCR. Similarly, the expression levels of 7 DEMs (novel-miR-107, novel-miR-116, novel-miR-176, novel-miR-194, novel-miR-381, novel-miR-513, and novel-miR-24) (Fig. 6C) and 7 DEGs (*pc1*, *mrtfb*, *fgf14*, *foxo1*, *celf1*, *cacna1*, and *dgkζ*) (Fig. 6D) from the identified miRNA-mRNA pairs between the AES and AE group were also assessed using RT-qPCR. The results demonstrated that the expression patterns of these DEMs and DEGs were consistent with the findings of the small RNA-Seq and RNA-Seq analyses.

## Discussion

MicroRNAs (miRNAs) are a group of non-coding endogenous small RNAs, typically 21–25 nucleotides in length, that play crucial roles in the response of fish to environmental stress [23–25]. Through interactions with target genes, miRNAs modulate cellular functions and metabolism by regulating gene expression levels [26]. Hence, it is highly significant to identify pivotal miRNAs associated with stress response in *C. nasus* and elucidate the underlying mechanisms involved in mediating stress response. MicroRNAs (miRNAs) are highly conserved molecules that selectively target mRNA to regulate gene expression [27, 28]. Previous studies have extensively documented the ability of miRNAs to regulate multiple target mRNAs, and reciprocally, mRNA can be regulated by multiple miRNAs [29, 30]. Numerous known miRNAs involving in various processes have been identified in different fish species under different types of stressors [23, 24]. However, in our study, we observed that most of the DEMs associated with glucose metabolism (novel-miR-240, novel-miR-241, novel-miR-176, novel-miR-194, etc.) and Ca^2+^ transport (novel-miR-381 and novel-miR-107) were novel miRNAs in the brains of *C. nasus* during air exposure and salinity adjustment. The unique spatial and temporal expression patterns of these novel miRNAs might be the key regulatory factors leading to stronger stress response in *C. nasus*. Although researches on novel miRNAs are relatively limited, growing numbers of evidence suggests their crucial role in gene regulation and biological processes [31–33]. Future investigations exploring the roles and functions of these predicted novel miRNAs in the brains of *C. nasus* during air exposure and salinity adjustment would be of great interest.

Elevation of blood glucose levels is a common response in fish experiencing stress. In this study, blood glucose significantly increased was in *C. nasus* during air exposure. Additionally, we identified several DEGs associated with glucose metabolism. Upregulated genes included *angptl7*, *g6p*, and *pepck*, while downregulated genes included *ir*, *5-ht7*, *sglt5*, and *glut4*. The decreased expression of insulin receptor (ir) led to reduced cellular sensitivity to insulin, resulting in insulin resistance, which inhibits glucose uptake and leads to elevated blood glucose levels [34]. Overexpression of *Angptl7* in healthy mice can also lead to insulin resistance-like characteristics, resulting in inhibited glucose uptake and insulin signal pathway [35]. g6p and pepck plays essential roles in gluconeogenesis, promoting glucose biosynthesis [36]. Blood glucose in fish plays a role in regulating stress responses. It is commonly observed that stress leads to elevated blood glucose levels in fish, which is primarily achieved through enhanced gluconeogenesis and reduced glucose transport mechanisms [37–41], which were similar to our results. Previous study proved that 10‰NaCl could reduce blood glucose levels in fish under transport stress [9–11]. In our study, we observed a decrease in blood glucose levels in *C. nasus* during salinity mitigation. Moreover, we identified several DEGs associated with glucose metabolism. Upregulated genes included *5-ht1e*, *glut4*, and *smct1*, while downregulated genes included *gr*, *dgkζ*, *g6p*, *celf1*, *foxo1*, *mrtfb*, and *fgf14*. Activated *foxo1* inhibited *glut4* transcription, leading to obstruction of glucose uptake [42]. *smct1* involved in glucose transport [43]. Up regulated *smct1* and *glut4* and down regulated *foxo1* in *C. nasus* during salinity mitigation indicated that glucose uptake was promoted. *foxo1* regulated gluconeogenesis by binding to promoter region of *g6p* [44]. dgkζ is an enzyme that phosphorylates diacylglycerol (DAG) to produce phosphatidic acid (PA). Inhibiting or reducing the production of PA can suppress glucose production in the liver [45]. These findings suggest that glucose biosynthesis is enhanced and glucose uptake is suppressed in *C. nasus* during air exposure, while the opposite trend is observed during salinity mitigation.

Organisms typically enhance their cellular energy metabolism under stress conditions to cope with challenges and stressors. Glucose serves as a crucial metabolic substrate that generates ATP and is metabolized via oxidative phosphorylation and the mitochondrial respiratory chain within mitochondria [46]. In our study, *NADH dehydrogenase* and *uqcc1* were upregulated, indicating activation of the mitochondrial respiratory chain for ATP generation in *C. nasus* during air exposure. However, this process also led to the production of high levels of reactive oxygen species (ROS), causing oxidative stress. Our results demonstrated that the antioxidative system was compromised, as evidenced by increased levels of MDA and LPO. Oxidative stress induced by ROS was triggered in fish responding to challenges and stress [47–49]. In contrast, during salinity mitigation, *NADH dehydrogenase*, *ndh*, and *nadpme* were upregulated, which activated the mitochondrial respiratory chain. This process also resulted in the production of plentiful ROS, but unlike air exposure, oxidative stress did not occur due to enhanced antioxidative activity. Additionally, the upregulation of *gpx* and *slc23a1* facilitated the production of GSH and transport of vitamin C, respectively. As common antioxidants, GSH and vitamin C can effectively remove ROS and improve antioxidative activity [50, 51]. These findings indicate that under air exposure, the excessive production of ROS by the mitochondrial respiratory chain disrupts the antioxidant system, resulting in oxidative stress in *C. nasus*. However, during salinity mitigation, the enhancement in antioxidative activity prevented oxidative stress in *C. nasus*.

Inflammatory responses are closely linked to oxidative stress, and ROS generated during oxidative stress play a critical role in sustaining inflammation [52, 53]. In our study, we observed upregulation in the expression of inflammation-related genes (*cod29*, *oit3*, *cysltr2*, *irak4*, and *blt1*) in *C. nasus* during air exposure. The activation of cd209, oit3, irak4, cysltr2, and blt1 can modulate the release of inflammatory factors through the activation of MAPK pathways, including p38 MAPK, ERK, and JNK, ultimately leading to inflammation [54–58]. Nevertheless, we observed a downregulation in the expression of *oit3*, *il-6rβ*, *traf6*, and *tnfrsf11* genes in *C. nasus* during salinity mitigation. The activation of IL-6Rβ is known to stimulate different members of the MAPK pathway, including ERK, p38 MAPK, and JNK [59–61]. In fish, the inflammatory response was primarily induced by the activation of MAPK signaling pathway, which was triggered by oxidative stress [62]. These findings suggested that inflammation occurred in *C. nasus* under air exposure, but is inhibited during salinity mitigation. Moreover, it is probable that the occurrence and suppression of inflammatory responses are regulated through the modulation of the MAPK signaling pathway.

The calcium signaling pathway is one of the important intracellular signaling pathways. As an important second messenger, changes of Ca^2+^ concentration can induce many physiological and pathological processes, which was regulated by calcium ion channels, calcium-binding proteins, and concentration gradients [63]. In the present study, expression of *nckx1*was decreased, while expression of *mcu* was increased. nckx1 is a transmembrane transport protein maintaining the balance of Ca^2+^ within cells via sodium-calcium exchange mechanism [64]. Increasing intracellular Ca^2+^ concentration activated mcu, inducing the influx of Ca^2+^ from the cytoplasm into the mitochondria. These entering Ca^2+^ can participate in regulating the activity of enzymes in the mitochondrial respiratory chain [65]. Increasing cortisol levels caused by stress can rapidly stimulate the increase of cytosolic free Ca^2+^ in rainbow trout (*Oncorhynchus mykiss*) [66]. These results suggested that the obstruction of Ca^2+^ transport to the outside cell increases the intracellular Ca^2+^ concentration, thus leading to an elevation in mitochondrial Ca^2+^ concentration in *C. nasus* under air exposure. However, expression of *cacna1i*, *trpv5*, *pc1*, and *calhm3* was down regulated, indicating that intracellular Ca^2+^ concentration was decreased via obstruction of Ca^2+^ transport to inside cell in *C. nasus* during salinity mitigation. Cytoplasmic Ca^2+^ overload may disturb the normal regulation of glucose metabolism pathways via affecting glucose metabolic enzyme activity, the structure and function of glucose transport proteins, and interfering with insulin signaling pathway [67–69]. Elevated levels of cytoplasmic Ca^2+^ increases mitochondrial Ca^2+^ concentration, leading to mitochondrial dysfunction. This disruption promotes the production of ROS within the mitochondria, inducing oxidative stress [70, 71]. Moreover, cytoplasmic Ca^2+^ overload can directly promote inflammatory responses and involve in the synthesis and release of multiple inflammatory factors [72]. Based on these findings, we speculate that the regulation of glucose metabolism, oxidative stress, and inflammation in *C. nasus* during air exposure could potentially be attributed to the cytoplasmic Ca^2+^ overload. Moreover, it is possible that the suppression of glucose metabolism, oxidative stress, and inflammation in *C. nasus* during salinity mitigation might be governed by the disruption of extracellular Ca^2+^ transport.

## Conclusion

In this study, substantial miRNA-mRNA regulation pairs were predicted, and hypothesized that they involved in glucose metabolism, Ca^2+^ transport, inflammation, and oxidative stress in *C. nasus* during air exposure and salinity mitigation. Interestingly, most of miRNAs associated with these processes were novel miRNAs. and this regulatory effect may differ from conservative regulatory relationships in other fish. Moreover, the miRNA-mRNA regulatory networks constructed in this study may regulate the increased/decreased plasma glucose and inhibited/promoted antioxidant activity during air exposure and salinity mitigation. Further researches on these novel miRNAs were necessary and would contribute to investigating the molecular mechanisms of response to air exposure and salinity mitigation in *C. nasus* and other fish.

## Materials and methods

### Air exposure stress

We conducted air exposure and salinity mitigation experiments using healthy *C. nasus* (11.10 ± 1.23 cm, 5.83 ± 1.67 g) obtained from the Freshwater Fisheries Research Center base in Yangzhong, China. The fish used in this study were 10 months old. Based on their gonads (transparent ribbon-like filaments), males and females were not able to be distinguished. We set the following three groups: a control group (C), an air exposure group (AE), and a 10‰ NaCl mitigation group (AES). Each group was replicated in triplicate, with 30 fish per tank measuring 75 cm × 56 cm × 60 cm. The experimental procedure is illustrated in Fig. 7. Briefly, the fish in the AE group and AES group were acclimated to freshwater and 10‰ NaCl (water temperature: 27.6 ± 0.7 ℃, pH: 7.8 ± 0.2, DO: 6.3 ± 0.9 mg/L), respectively, for two weeks prior to air exposure. After air exposure by netting [8, 73], the fish in the AE and AES groups were returned to freshwater and 10‰ NaCl, respectively, for 30 min of recovery. Subsequently, five fish were chosen at random and anesthetized using 50 mg/L MS-222. The brain tissues were rapidly collected and flash-frozen in liquid nitrogen, then stored at -80℃ until further analysis. Blood samples were incubated at 4℃ for 2 h, followed by centrifugation at 3500 r/min for 10 min at 4℃ to obtain serum.

### Detection of biochemical indexes

Brain tissue stored at -80 ℃ was weighed accurately. The brain tissue was homogenized in nine volumes of normal saline. The homogenate was centrifuged at 250 r/min for 10 min. Then the supernatant (10% tissue homogenate) was taken for further analysis. The levels of serum glucose (GLU), CAT, SOD, GSH-Px, T-AOC, MDA, and LPO in brain tissues were measured using kits obtained from Jiancheng (Nanjing, China) following the manufacturer’s instructions.

### RNA extraction

Total RNA was isolated from 9 brain tissues using TRIzol® Reagent following the manufacturer’s instructions (Invitrogen, CA, USA), and DNase I treatment (TaKara, Tokyo, Japan) was employed. After ensuring quality and quantification, high-quality RNA samples (OD260/280 = 1.8 ∼ 2.2, OD260/230 ≥ 2.0, RIN ≥ 6.5, 28 S:18 S ≥ 1.0, > 10 µg) were used for constructing the sequencing libraries.

### microRNA libraries construction and sequencing

Small RNA-seq libraries were generated using the TruSeq Small RNA Sample Preparation Kit from Illumina (San Diego, CA). 5µg RNA was used for library preparation. The total RNA was subjected to polyacrylamide gel electrophoresis (PAGE) to separate it into different fragment sizes. Small RNA fragments within the range of 18 ∼ 30 bp were preferentially selected. To generate cDNA constructs, reverse transcription followed by PCR was utilized using small RNA molecules ligated with 3’ and 5’ adapters. The cDNA was purified and recovered using PAGE gel, and the resulting product was dissolved in EB solution for subsequent labeling. Paired-end libraries were sequenced on the Illumina NovaSeq 6000 sequencing platform.

The raw single-end reads underwent trimming and quality control using Trimmomatic with default parameters. Additionally, fastx_clipper was employed to remove the adaptor sequence (TGGAATTCTCGGGTGCCAAGG). To eliminate common RNA families and repeats, the adapter-removed small RNA sequences were aligned to the Rfam/RepBase database. Following that, unique sequences ranging from 18 to 26 bp were aligned to miRBase 22.0 using a BLAST search to identify known and novel miRNAs. Known miRNAs were identified as unique sequences that aligned to the hairpin arms of mature miRNAs from specific species. The unique sequences that aligned to the opposing arm of the annotated mature miRNAs within the precursor hairpin of specific species were regarded as potential candidates for novel 5p- or 3p-derived miRNAs. Both the known miRNAs identified in the hairpin arms and the miRNAs determined through this process were classified as known miRNAs. The unmapped sequences were analyzed through a BLAST search against specific genomes, and hairpin RNA structures containing these sequences were predicted using the RNAfold software, with consideration of the flanking 80 nt sequences. (http://rna.tbi.univie.ac.at/cgi-bin/RNAfold.cgi). Differentially expressed analysis of miRNAs based on normalized deep-sequencing counts was performed using ANOVA. The significance set at *P*-value < 0.05. To TargetScan 5.0 and Miranda 3.3a were employed to identify miRNA binding sites to predict the genes targeted by miRNAs. Then the overlaps of predictions from both algorithms were combined. Additionally, Gene Ontology (GO) terms and KEGG Pathway annotations were assigned to the most abundant miRNAs and their target genes.

### RNA-Seq libraries construction and sequencing

TruSeqTM RNA sample preparation Kit from Illumina (San Diego, CA) was used for RNA-seq libraries construction. 1.0 µg of total RNA was used for library preparation. According to Illumina’s protocol, isolating mRNA, synthesizing cDNA, end repair, adding A-base, and ligation of the Illumina-indexed adaptors were carried out. The libraries obtained were subjected to size selection on a 2% Low Range Ultra Agarose gel to isolate cDNA fragments within the range of 200 to 300 bp. Subsequently, PCR amplification was performed using Phusion DNA polymerase (NEB, Beijing, China) for 15 PCR cycles. Quantificational paired-end libraries were sequenced on the Illumina NovaSeq 6000 sequencing platform. After trimming and quality control of raw reads using Trimmomatic (version 0.36), the generated clean reads were mapped to the *C. nasus* reference genome (GCA_007927625.1) using hisat2 software. qualimap_v2.2.1 was used for the quality assessment of the mapped reads. Gene read counts were obtained using htseq. The R statistical package edgeR was used for differentially expressed analysis according to the fragments per kilobase of exon per million mapped reads (FPKM). Genes with foldchange > 2.0 and false discovery rate (FDR) < 0.05 were considered as differentially expressed genes (DEGs). To gain insights into the functions of the DEGs, GO and KEGG enrichment were performed using Goatools and KOBAS. Bonferroni-corrected *P*-value < 0.05 was considered as significant enrichment.

### Integrated analysis of the DEMs and DEGs data

TargetScan v5.0, miRanda v3.3a, and TargetFinder were utilized to perform prediction of the genes targeted by DEMs. Based on compared predicted target genes with the DEGs and the negative regulation of miRNA and mRNA, pairs of negatively correlated DEMs and target DEGs were identified. The pairs of negatively correlated DEMs and target DEGs were visualized using a Sankey diagram through the OmicShare Tools platform (https://www.omicshare.com/tools/). Furthermore, considering the functions and negative regulation of the DEMs and target DEGs, a regulatory network of miRNA-mRNA interactions in *C. nasus* brains during air exposure and salinity mitigation was constructed and visually presented using Adobe Illustrator CS6 software (San Jose, USA).

### RT-qPCR validation

To verify the accuracy of small RNA and transcriptome sequencing data, 12 DEMs and 12 DEGs were detected via RT-qPCR. The primers designed by Primer Premier 5.0 software were shown in Table S9 for RT-qPCR validation. Mir-X™ miRNA First- Strand Synthesis and TB Green® qRT-PCR User Manual (Takara, Beijing, China) was employed for miRNA quantitative detection following the manufacturer’s instructions. RT-qPCR were detected on Bio-Rad CFX96 real-time PCR system (Bio-Rad, Hercules, CA, USA) following the directions [74]. The relative expression of DEMs and DEGs was calculated using the log_2_ foldchange 2^−ΔΔCt^ method (*n* = 9) [75], using *β-actin*, and *U6 snRNA* for the reference genes [76].

### Statistical analysis

To evaluate the statistical significance, we conducted a one-way analysis of variance (ANOVA) with the Duncan test using SPSS 20 software. The data were expressed as mean ± standard deviation (SD). Statistical significance was considered at a significance level of *P* < 0.05.

### Electronic supplementary material

Below is the link to the electronic supplementary material.

Supplementary Material 1

## Data Availability

The miRNA sequences were submitted to NCBI SRA database (PRJNA1018690, https://www.ncbi.nlm.nih.gov/bioproject/PRJNA1018690). The mRNA sequences were submitted to NCBI SRA database (PRJNA1018811, https://www.ncbi.nlm.nih.gov/bioproject/PRJNA1018811).
