# Supplementary figures and images for: Integrated mRNA and miRNA analysis reveals the regulatory network of oxidative stress and inflammation in Coilia nasus brains during air exposure and salinity mitigation
Source: BMC Genomics. 2024 May 7;25:446. doi: 10.1186/s12864-024-10327-w (PMC11075292; doi:10.1186/s12864-024-10327-w)

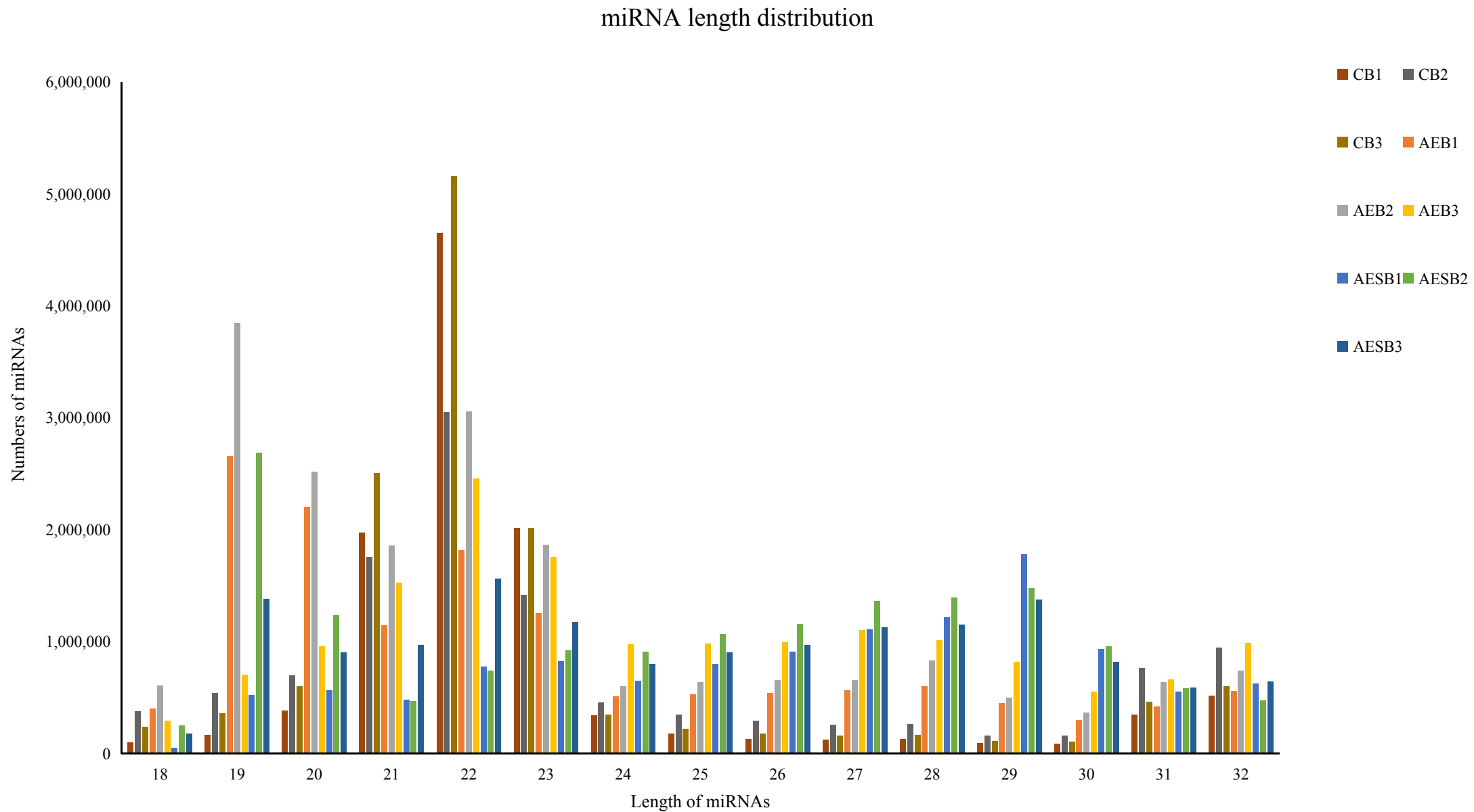

Figure S1. miRNA length distribution in the present study.

Supplement: Supplementary file 1 — Supplementary Material 1 [file 12864_2024_10327_MOESM1_ESM.zip › supplymentary materials/supplymentary figure.pdf]
